# Effect of Water-Glass Coating on HA and HA-TCP Samples for MSCs Adhesion, Proliferation, and Differentiation

**DOI:** 10.1155/2016/9758729

**Published:** 2016-06-26

**Authors:** Indu Bajpai, Duk Yeon Kim, Jung Kyong-Jin, In-Hwan Song, Sukyoung Kim

**Affiliations:** ^1^Materials Science and Engineering, Yeungnam University, 280 Daehak-Ro, Gyeongsan, Gyeongbuk 712-749, Republic of Korea; ^2^Department of Anatomy, College of Medicine, Yeungnam University, 317-1 Daemyung-Dong, Daegu 705-717, Republic of Korea

## Abstract

Ca-P and silicon based materials have become very popular as bone tissue engineering materials. In this study, water-glass (also known as sodium silicate glass) was coated on sintered hydroxyapatite (HA) and HA-TCP (TCP stands for tricalcium phosphate) samples and subsequently heat-treated at 600°C for 2 hrs. X-rays diffraction showed the presence of *β*- and *α*-TCP phases along with HA in the HA-TCP samples. Samples without coating, with water-glass coating, and heat-treated after water-glass coating were used to observe the adhesion and proliferation response of bone marrow derived-mesenchymal stem cells (MSCs). Cell culture was carried out for 4 hrs, 1 day, and 7 days. Interestingly, all samples showed similar response for cell adhesion and proliferation up to 7-day culture but fibronectin, E-cadherin, and osteogenic differentiation related genes (osteocalcin and osteopontin) were significantly induced in heat-treated water-glass coated HA-TCP samples. A water-glass coating on Ca-P samples was not found to influence the cell proliferation response significantly but activated some extracellular matrix genes and induced osteogenic differentiation in the MSCs.

## 1. Introduction

Calcium-phosphate (Ca-P) based materials, in either single phase or multiphase, are well known for their bioactivity as a bone substitute [1, 2]. Hydroxyapatite (HA), tricalcium phosphate (TCP), and their different compositions are widely used Ca-P ceramics in the biomedical field [3]. On the other hand, bioactive glasses have been also used in various forms [4]. Herein, presence of silicon (Si) ions was found to influence the bioactivity of other materials in various ways [5]. The combination of Si with Ca and P ions has reported to enhance the apatite formation and affect some cellular functions [6, 7].

Porter et al. found the different types on apatite formation on the HA and Si substituted-HA (Si-HA) granules in a mouse model. However, Si-HA showed rapid bone remodeling than the pure HA [8]. Obata et al. observed the better effect of silica layer on the in vitro proliferation of the osteoblast-like cells (MC3T3-E1) and apatite formation on the titanium surface as compared to hydroxyapatite [9]. Padilla et al. investigated better apatite deposition and proliferation of human osteoblastic-like cell line in the mixture of HA and sol gel glass than the HA alone [10]. Lopes et al. found better bone and implant contact in tibia of rabbits in the case of glass-reinforced-HA as compared to monolithic HA [11]. There are several more studies that showed the better biological response of glass containing Ca-P samples as compared to Ca-P alone [12]. Our research group have also reported the improvement in apatite formation by a water-glass coating on porous Ca-P scaffolds [13].

Human bone marrow-derived-mesenchymal stem cells (MSCs) are the primary cultured cells which have self-replication ability and can differentiate in other types of cells according to surrounding conditions [14, 15]. In addition, extra cellular matrix (ECM) and differentiation related several important genes are marked in MSCs [16, 17]. MSCs are being used in the orthopedic applications because of their differentiation property on the Ca-P ceramic substrates along the osteogenic pathway [18, 19]. Therefore, MSCs are selected as the suitable cellular model for the study of adhesion, proliferation, and differentiation on the ceramic samples.

The aim of current study is to observe the effect of water-glass (WG) coating on Ca-P samples in terms of cell adhesion, proliferation, and differentiation. In this study, water-glass (WG) which is known as sodium-silicate glass was coated on HA and BCP (which is abbreviation used for HA-TCP sample) samples followed by heat treatment. This is a comparative study presenting the different cellular functions influenced by differently treated substrate surfaces.

## 2. Materials and Methods

Commercially available hydroxyapatite (HA), dicalcium phosphate dehydrate (DCPD), and Ca(OH)_2_ were used as raw materials to prepare samples. In order to fabricate the HA-TCP (BCP) samples, 100 grams DCPD, 100 grams HA, and 21 grams Ca(OH)_2_ were mixed by wet ball milling in ethanol medium for 1 day. Further, mixed powder was dried in oven at 80°C temperature. Green pallets of 15 mm diameter of HA and above mixture were prepared at 45 MPa pressure and all samples were sintered at 1250°C temperature for 2 hrs with the 5°C/min heating rate [20].

All sintered samples of HA and HA-TCP were cleaned in ethanol by ultrasonication and then dried and coated with 10 vol% water-glass by spin coater at 200 rpm. One set of water-glass coated samples was heat-treated at 600°C for 2 hrs. Description of samples used in current study is written in Table 1. This study is based on comparisons of cell behavior on six different sets of samples; (1) HA, (2) HA with WG coating, (3) HA with WG coating and heat treatment, (4) BCP, (5) BCP with WG coating, and (6) BCP with WG coating and heat treatment.

## 3. Characterizations

Phase analysis of all the samples was conducted by X-ray diffractometer (XRD, Rigaku) on 40 KV and 30 mA current with 1 deg/min scan rate in the 2-theta range of 25°–60° and recorded XRD patterns were analyzed by using the X'Pert HighScore Plus Software. In order to quantify the phase composition from the XRD patterns, Rietveld mode associated with X'Pert HighScore Plus software was used.

## 4. Cell Culture

We have previously described that human bone marrow derived-mesenchymal stem cells were isolated from the aspirated iliac crest of patients undergoing nonemergency orthopedic surgery through an Internal Review Board approved protocol at Yeungnam University Hospital using a previous method [20, 21]. The experimental cells were cultured in the culture media that is DMEM medium supplemented with 10% FBS (FBS; Gibco-BRL, Rockville, MD) and 1% antibiotic-antimycotic (Gibco) at 37°C with 5% CO_2_. In this study, the second passages cells were used for the experiments. For the cell culture, all samples were sterilized by ethylene oxide gas. Prior to cell incubation, all samples were placed in the culture media for 4 hrs for media adaptation and then MSCs were seeded in the fresh culture media.

### 4.1. Immunofluorescence Analysis

In order to observe the adhesion and proliferation of MSCs, all samples were seeded with a cell density of 3 × 10^4^/cm^2^ and incubated for 4 hrs, 1 day, and 7 days at 37°C in biological incubator. To image the cells under fluorescence microscopy, all incubated samples were washed three times with 1x PBS followed by fixation with 3.7% formaldehyde for 20 min. Further fixed cells were washed and permeabilized with 0.1% Triton X-100 for 10 min and background blocking was done by incubation for 30 min in a 0.1% bovine serum albumin (BSA) solution. Subsequently, all samples were stained with a 1 : 40 dilution fluorescein-conjugated phalloidin (Invitrogen, Carlsbad, CA, 200 U/mL) solution for 1 hr for the staining of actin fibers. After that, nuclei of the cells were stained by using a 100 nM 4′-6-diamidino-2- phenylindole (DAPI, Sigma) solution. Finally, washed samples were mounted using anti-fluorescence-fade medium (1,4-Diazabicyclo[2.2.2]octane, DABCO, Sigma) and observed under fluorescence microscopy (Leica DM6000B, Germany). The detailed protocol of cell staining has been also described in our previous work [20]. For the quantification of the cell adhesion and proliferation on the different samples, minimum of five images of the nuclei of the cells were taken at different areas of the samples and the number of cells/unit area for each sample was plotted in Figure 3.

### 4.2. Reverse Transcription Polymerase Chain Reaction (RT-PCR)

As previously we described that to measure the expression levels of the ECM genes, the total RNA was isolated from human bone marrow derived-mesenchymal stem cells grown on the substrates used in current study, in 100 *μ*L of TRIzol® Reagent (Ambion by Life Technologies, Gaithersburg, MD) reagent [20]. 20 *μ*L of chloroform was mixed with the sample and the solution was incubated for 2 min at room temperature. The samples were then centrifuged at 12,000 rpm for 15 min at 4°C. Next, 50 *μ*L of the upper aqueous phase was carefully transferred to a fresh tube and the RNA was precipitated by mixing with 50 *μ*L isopropyl alcohol. The solution was incubated for 1 hour at −20°C and centrifuged at 12,000 rpm for 15 min at 4°C. The supernatant was removed and the RNA pellet was washed once with 70% ethanol. The RNA pellet was air-dried and dissolved in diethylpyrocarbonate-treated water (Amresco, USA). The cDNA was synthesized using an oligo (dT) primer (Genotech, South Korea) and M-MLV reverse transcriptase (Promega, Fitchburg, WI) following the manufacturer's instructions. The results were normalized to those for human actin. The primers and PCR cyclic conditions for each gene are presented in Table 2.

## 5. Results and Discussion

**Figure 1:**
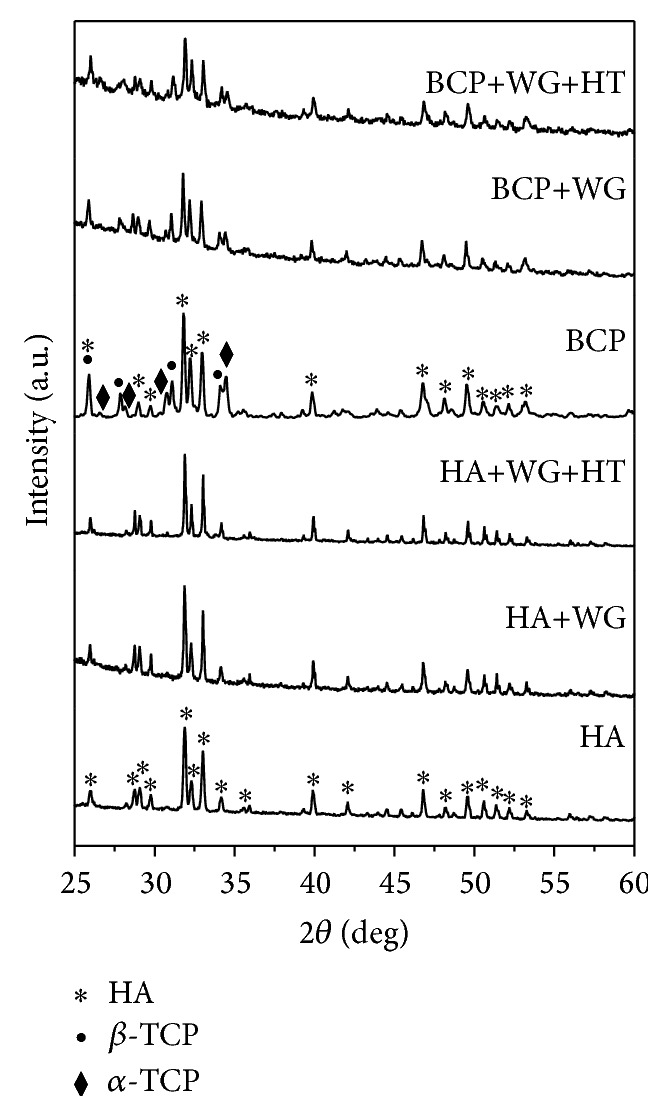
XRD analysis of the water-glass coated Ca-P samples.

Figure 1 shows the XRD analysis of water-glass coated HA and BCP samples. XRD peaks of sintered HA commensurate well with the peaks of standard HA (ICSD code 157481). However, water-glass coated and heat treatment of water-glass coated samples at 600°C did not reveal presence of any addition phase along with HA. The critical analysis of XRD patterns of the BCP sample showed the presence of *β*-TCP and *α*-TCP along with the HA phase. The quantification of these three phases was obtained by X'Pert HighScore operated in Rietveld mode which showed 56%  HA, 25%  *β*-TCP, and 19%  *α*-TCP phases in the BCP samples. In addition, the effect of heat treatment of water-glass coating on BCP samples was similar to HA. All sintered HA and BCP samples are nearly fully densified.

**Figure 2:**
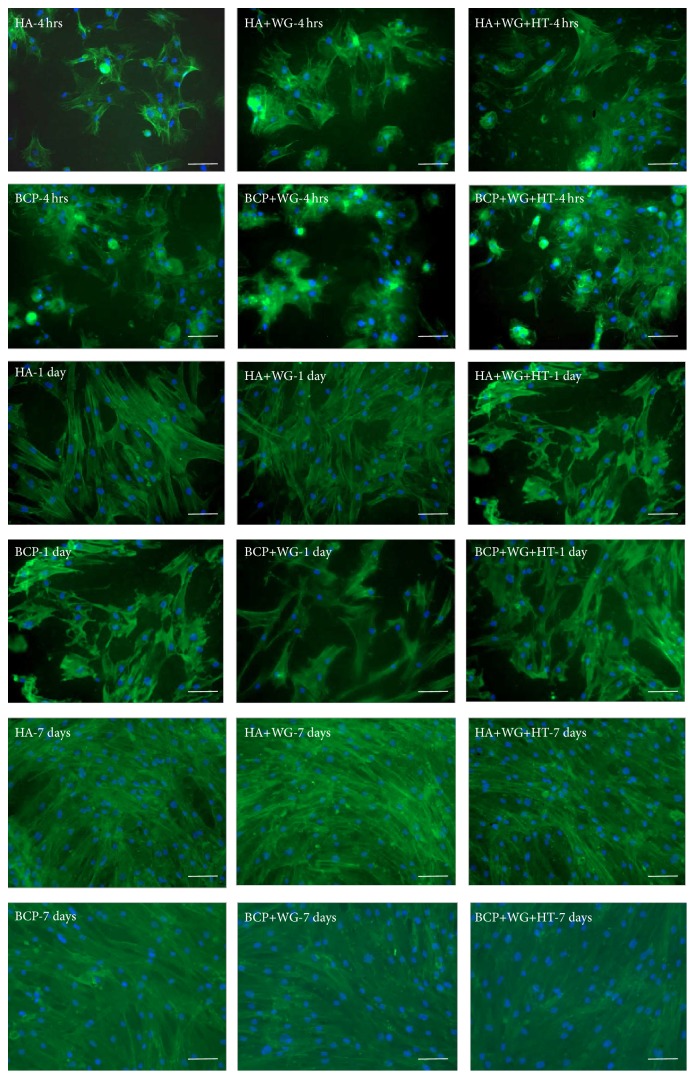
Fluorescence microscopy images of MSCs cultured on different water-glass coated Ca-P samples for 4 hrs, 1 day, and 7 days. Scale bar = 100 *μ*m.

Figure 2 presents the fluorescence microscopy images of MSCs, stained with DAPI and FITC-conjugated phalloidin, seeded on WG coated Ca-P samples for 4 hrs, 1 day, and 7 days. Herein, 4 hrs culture was done to observe the adhesion property of cells on these samples and all samples showed good cell adhesion. After 1 day all attached cells spread properly on the surface. Further, cells on all samples proliferated well and almost reached confluence after 7-day incubation.

**Figure 3:**
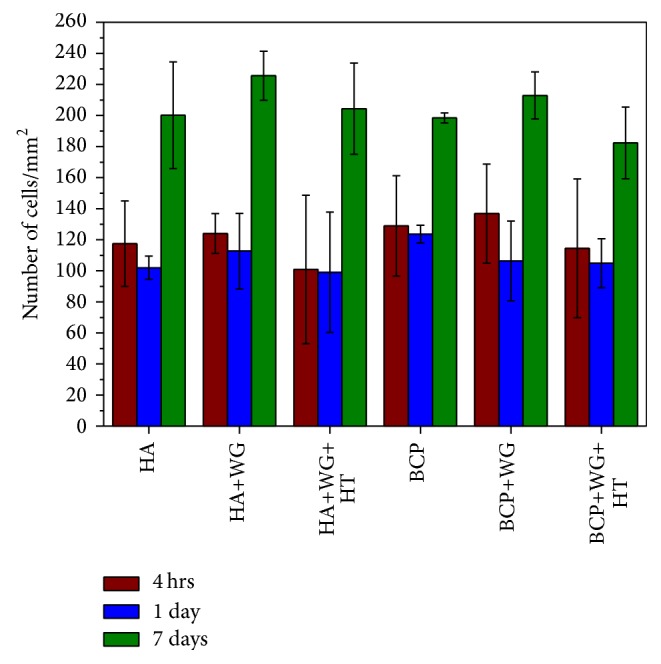
Adhesion and proliferation of cells on water-glass coated Ca-P samples when cultured for 4 hrs, 1 day, and 7 days. Statistically significant difference was not observed among the samples incubated for same duration.

Figure 3 shows the number of cells/unit area on each sample after 4 hrs, 1 day, and 7-day incubation. After culture, the attached cells were stained with DAPI, the cell nuclei were counted manually, and the number of cells/unit areas was plotted for the each sample. Average cell density of 1-day incubation is decreased as compared to 4 hrs but there is no statistically significant difference in data. The 4 hrs cell incubation shows the initial attachment of cells which is not very strong on ceramic sample. It is possible that seeding solution contains nonhealthy cells as well and those were also initially attached on the sample surface but died later on. Although all the samples showed similar cell adhesion and proliferation after 7-day culture WG coated HA and BCP samples showed slightly higher but not statistically significant difference in cell density as compared to noncoated samples and coated-heat-treated samples.

**Figure 4:**
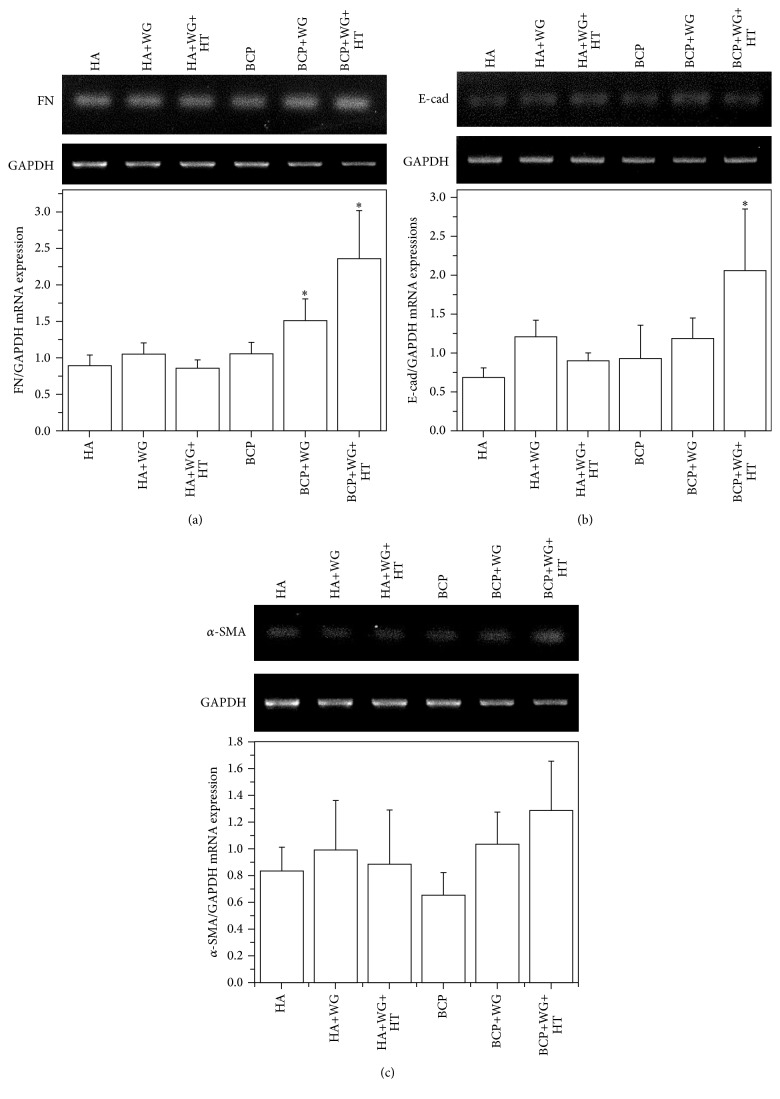
ECM-related gene expressions of MSCs when cultured on water-glass coated Ca-P samples for 7 days. The values are expressed in mean ± SE. (*∗*) denotes a significant difference (*p* < 0.05) between the samples as compared to HA for *n* = 3.

To determine the interaction between the cells and the materials, extracellular matrix- (ECM-) related genes expression, including fibronectin (FN) (Figure 4(a)), E-cadherin (E-cad) (Figure 4(b)), and *α*-smooth muscle actin (*α*-SMA) (Figure 4(c)), were investigated after 7-day culture. Fibronectin binds the extra cellular matrix components and plays key role in adhesion dependent cell growth, proliferation, signaling event, and differentiation. FN expression was induced significantly in the cells grown on the BCP+WG+HT sample but not significantly different than the BCP+WG. On the other hand, HA, HA+WG, and HA+WG+HT showed no statistically significant difference in FN expressions.

E-cad is transmembrane protein which is very important for cell adhesion and Ca^+2^ ions are the main source of this gene to function [22]. In addition, E-cad shows the calcium dependent cell adhesion [23]. An E-cad expression was observed significantly in BCP+WG+HT sample only. Similar E-cad expressions were observed in other five samples. *α*-SMA is important for cell molality, integrity, and proliferation and the induced values corresponding to this gene are presented in Figure 4(c) which showed no significant difference among the samples.

**Figure 5:**
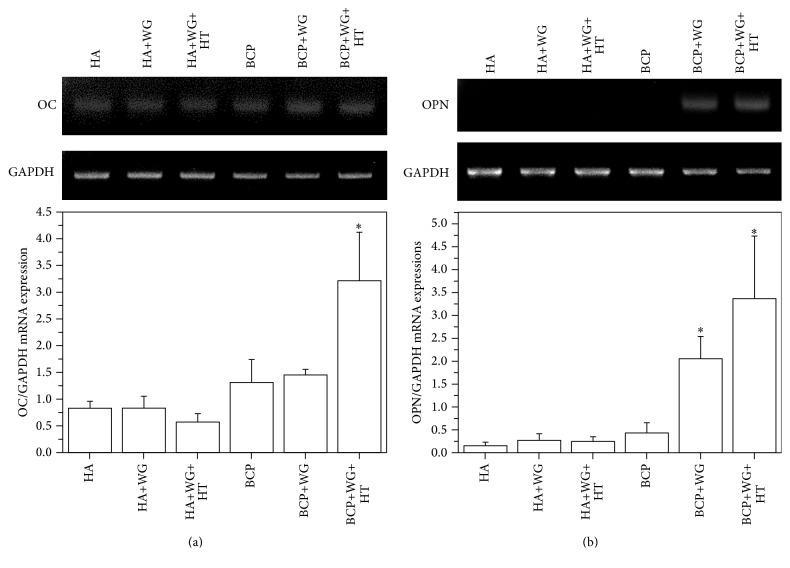
Osteogenic differentiation related gene expressions of MSCs when cultured on water-glass coated Ca-P samples for 7 days. The values are expressed in mean ± SE. (*∗*) denotes a significant difference (*p* < 0.05) between the samples as compared to HA for *n* = 3.

Figure 5 presents the osteogenic differentiation related gene (osteocalcin (OC) and osteopontin (OPN)) expressions. Osteocalcin is a noncollagenous protein found in bone matrix which regulates mineralization and calcium ions homeostasis [24]. Herein, OC gene expressions were significantly induced in BCP+WG and BCP+WG+HT as shown in Figure 5(a).  Figure 5(b) presents the values corresponding to OPN gene which is a multifunctional highly negatively charged protein and found in mineralized extra cellular matrix of bone [25]. Current study showed the significant OPN expressions in cells grown on BCP+WG and BCP+WG+HT samples. These results reveal that the water-glass coating activated the osteogenic differentiation in the MSCs and it is higher on BCP based samples as compared to HA-based samples.

Although the dissolution test data is not presented in this study, according to the available scientific literature, dissolution of BCP is higher than the pure HA [20]. In BCP the dissolution of calcium is faster than phosphorous and water-glass releases the soluble Si ions [9]. In addition, heat treatment of water-glass controls the dissolution of Si ions in the culture medium and increases the durability of the coating layer. On the other hand, the proper dissolution of ions (calcium, phosphorous, and silicon) in culture media to induce the osteogenic differentiation is still not specified.

The current study presents the effect of water-glass coating on the gene expressions of the MSCs when cultured on Ca-P samples. Although the significant difference in the proliferation of MSCs was not observed up to 7-day culture some genes were affected by water-glass coating on the Ca-P samples. This indicates that the released Si-ions from the water-glass coating participated in the cellular activation in vitro. However, uncertainty remains about the mechanism by which water-glass induced the FN, E-cad, OPN, and OC gene expressions in MSCs. Results of the current study show that the gene expression corresponding to mineralization of the cell matrix was significantly induced by the water-glass coating. Some studies have shown the significant cell proliferation because of the presence of the Si ions in the culture medium [26] but in the current study we could not observe the significantly higher cell proliferation but average value was slightly higher on WG coated HA and BCP samples. Cell material interaction results might not be similar in all the cases because this interaction also depends on the type of used cells for the experiments. Therefore, long term cell culture studies are required for the more understanding of the effect of the water-glass coating on the cell response.

Several studies have reported the in vivo and in vitro effect of presence of Si along with Ca-P on the cell proliferation and new bone formation but little is known about the influence of water-glass on gene activation [27, 28]. An in vitro study reported that cell cycle of the human osteoblast was controlled and genes were activated by the dissolution of soluble ions from bioactive glass substrates [7]. However, gene activation because of Si and Ca^2+^ ions released from bioactive glass was considered responsible for leading fast new bone formation in vivo [8]. Therefore, it can be considered as the released Si ions in the culture media along with Ca and P ions can activate the several genes in the MSCs.

The current study also shows the activation of osteogenic differentiation related genes (OC, ONP) by a water-glass coating on the Ca-P substrates and this effect is higher on water-glass coated-BCP samples as compared to water-glass coated-HA sample because of faster release of Ca^2+^ ions from the BCP samples.

## 6. Conclusions

This study describes the effect of a water-glass coating on Ca-P substrates in terms of adhesion, proliferation, and ECM- and osteogenic differentiation- related gene expression of MSCs. The cell adhesion and proliferation showed no statistical significant difference between the samples regardless of water-glass coating. On the other hand, BCP+WG+HT showed the significant ECM- and osteogenic differentiation- related gene expression (FN, E-cad, OC, and OPN) while remaining similar to other samples in case of *α*-SMA. In addition, all water-glass coated-BCP samples showed higher values of gene expressions than the water-glass coated-HA samples because of the faster dissolution of BCP as compared to HA, in the culture media. Therefore, it can be considered as the released Si ions (from water-glass coating) in the culture media along with Ca and P ions (from BCP samples) can activate the several genes and induce the osteogenic differentiation in the MSCs.

## Figures and Tables

**Table 1:** Sample's details and ID.

	Sample ID	Sample details
1	HA	Pure sintered HA
2	HA+WG	Sintered HA coated with water glass
3	HA+WG+HT	Heat treated water glass coated sintered HA
4	BCP	Sintered HA-TCP (*β*-TCP + *α*-TCP) composite
5	BCP+WG	Water glass coated sintered BCP
6	BCP+WG+HT	Heat treated water glass coated sintered BCP

**Table 2:** Primers and PCR conditions.

	Sense (5′-3′)	Antisense (5′-3′)	Annealing/cycle
Fibronectin (FN)	AGT TCA GGG TTC CTG GAA	TGC CAC TGT TCT CCT ACG TG	56°C/25
E-cadherin (E-cad)	CAA GGA CAG CCT TCT TTT CG	TGG ACT TCA GCG TCA CTT TG	57°C/38
Osteocalcin (OC)	CCC TCA CAC TCC TCG CCC TAT	TCA GCC AAC TCG TCA CAG TCC	59°C/30
Osteopontin (OPN)	CCA AGT AAG TCC AAC GAA AG	GGT GAT GTC CTC GTC TGT A	57°C/30
*α*-smooth muscle actin (*α*-SMA)	ACT GTG TTA TGT AGC TCT GGA C	ACA ATG GAA GGC CCG GCT TC	57°C/25
GAPDH	AGG TCG GAG TCA ACG GAT TTG	GTG ATG GCA TGG ACT GTG GT	58°C/25
